# Practical strategies to reduce nosocomial transmission to healthcare professionals providing respiratory care to patients with COVID-19

**DOI:** 10.1186/s13054-020-03231-8

**Published:** 2020-09-23

**Authors:** Ramandeep Kaur, Tyler T. Weiss, Andrew Perez, James B. Fink, Rongchang Chen, Fengming Luo, Zongan Liang, Sara Mirza, Jie Li

**Affiliations:** 1 Division of Respiratory Care, Department of Cardiopulmonary Sciences, Rush University Medical Center, 1620 W Harrison St, Tower LL1202, Chicago, IL 60612 USA; 2 Shenzhen Institute of Respiratory Disease, Shenzhen People’s Hospital (First Affiliated Hospital of South University of Science and Technology of China), Shenzhen, China; 3 Department of Respiratory and Critical Care Medicine, West China Medical Center of Sichuan University, Chengdu, China

**Keywords:** Nosocomial infection, Respiratory care, Aerosol-generating procedures

## Abstract

Coronavirus disease (COVID-19) is an emerging viral infection that is rapidly spreading across the globe. SARS-CoV-2 belongs to the same coronavirus class that caused respiratory illnesses such as severe acute respiratory syndrome (SARS) and Middle East respiratory syndrome (MERS). During the SARS and MERS outbreaks, many frontline healthcare workers were infected when performing high-risk aerosol-generating medical procedures as well as when providing basic patient care. Similarly, COVID-19 disease has been reported to infect healthcare workers at a rate of ~ 3% of cases treated in the USA. In this review, we conducted an extensive literature search to develop practical strategies that can be implemented when providing respiratory treatments to COVID-19 patients, with the aim to help prevent nosocomial transmission to the frontline workers.

## Introduction

Coronavirus disease (COVID-19) cases were first reported to the World Health Organization on December 31, 2019 [1]. Since then, this illness has spread exponentially in over 200 countries. As of June 9, 2020, there were 7,039,918 confirmed cases of the COVID-19 disease globally [2]. Even though the exact mode of COVID-19 transmission has been debatable, the route of COVID-19 transmission is reported to be from person-to-person contact and exposure to respiratory droplets (> 5–10 μm) [3], whereas airborne transmission (< 5 μm) during aerosol-generating procedures remains under investigation [4, 5]. Based on the initial data reported [6–12], around 5–30% of COVID-19 patients develop signs of severe respiratory distress requiring intensive care unit (ICU) admission to receive advanced respiratory support in terms of oxygen therapy, non-invasive and invasive ventilatory support with prone positioning (Table 1).

**Table 1:** Use of respiratory interventions in COVID-19 patient population

Study	Huang et al. [5] (***n*** = 41)	Wu et al. [6] (***n*** = 201)	Wang et al. [7] (***n*** = 138)	Guan et al. [8] (***n*** = 1099)	Yang et al. [9] (***n*** = 52)	Arentz et al. [10] (***n*** = 21)	Grasseli et al. [11] (***n*** = 1591)	Richardson et al. [12] (***n*** = 5700)
**Study design**	Prospective cohort, single center	Retrospective cohort, single center	Retrospective, single-center case series	Retrospective data from 552 hospitals in China	Retrospective cohort, single center	Retrospective cohort, single center	Retrospective case series	Retrospective case series
**Study population**	Confirmed COVID-19 cases from Dec 16, 2019, to Jan 2, 2020	Confirmed COVID-19 cases from Dec 25, 2019, to Jan 26, 2020	Confirmed COVID-19 cases from Jan 1 to Jan 28, 2020	Confirmed COVID-19 cases through January 29, 2020	Critically ill^b^ confirmed COVID-19 cases from late Dec 2019 to Jan 26, 2020	Confirmed COVID-19 cases admitted to ICU from Feb 20 to March 5, 2020	Critically ill laboratory-confirmed COVID-19 cases from Feb 20 to March 18, 2020	Confirmed COVID-19 cases admitted to 12 hospitals in New York City from March 1 to April 4, 2020
**Age**	49 (IQR, 41–58)	51 (IQR, 43–60)	56 (IQR, 42–68)	47 (IQR 35–58)	59.7 (± 13.3)	70 (IQR, 43–92)	63 (IQR, 56–70)	63 (IQR, 52–75)
**ICU admission**	13 (32%)	53 (26.4%)	36 (26%)	55 (5%)	52 (100%)	21 (100%)	1591 (100%)	373/2634 (14.2%)
**Acute respiratory distress syndrome**	12 (29%)	84 (41.8%)	27 (19.6%)	37 (3.4%)	35 (67%)	20 (95%)	NR	NR
**Oxygen therapy**	27 (66%)	98 (49%)	106 (77%)	454 (41.3%)	NR	NR	13/1300 (1%)	1584/5693 (27.8%)
**High-flow nasal cannula**	10 (24%)^a^	NR	NR	NR	33 (63.5%)	1 (4.8%)	NR	NR
**Non-invasive ventilation**	10 (24%)^a^	61 (30%)	15 (10.9%)	56 (5.1%)	29 (56%)	4 (19%)	137/1300 (11%)	NR
**Invasive mechanical ventilation**	2 (5%)	5 (2.5%)	17 (12.3%)	25 (2.3%)	22 (42%)	15 (71%)	1150/1300 (88%)	320/2634 (12.2%)
**Prone position ventilation**	NR	NR	NR	NR	6 (11.5%)	8 (38%)	240/875 (27%)	NR
**ECMO**	2 (5%)	1 (0.5%)	4 (2.9%)	5 (0.5%)	6 (11.5%)	NR	5/498 (1%)	NR

*Abbreviations*: *ICU* intensive care unit, *IMV* invasive mechanical ventilation, *ECMO* extracorporeal membrane oxygenation, *NR* not reported

^a^Reported as NIV or HFNC use; ^b^defined as those admitted to ICU requiring mechanical ventilation or had FiO_2_ ≥ 0.6

Standard droplet and contact precautions (gowns, gloves, mask) are known to reduce the risk of contracting severe acute respiratory syndrome (SARS) [13] but not under all circumstances, especially when performing high-risk procedures such as intubation [14]. A recent systematic meta-analysis showed that a physical distance of 1 m or more and wearing a mask is optimum to reduce person-to-person virus transmission and to keep healthcare workers (HCWs) from contracting the SARS-CoV-2 infection [15]. During the SARS outbreak, many frontline HCWs were infected via nosocomial transmission due to failure to implement adequate infection control precautions, especially when performing aerosol-generating medical procedures (AGMPs) [16–18], such as bronchoscopy, intubation, suctioning, invasive and non-invasive ventilation (NIV), bag mask ventilation, and nebulization [19–21]. In a prospective study, Macintyre et al. [22] reported that clinicians who performed AGMPs were at greater risk of acquiring the infection as compared to those who were not involved in such procedures [adjusted relative risk (RR) 2.90, 95% confidence interval (CI) 1.42–5.87]. Considering SARS-CoV-2 belongs to the same family as SARS, frontline clinicians delivering AGMPs to COVID-19 patients are likely at a similar high risk of transmission and infection. According to the Centers for Disease Control and Prevention (CDC), 95,860 (incidence of 3%) HCWs have been reported to be infected with COVID-19 in the USA, with at least 515 deaths as of July 10, 2020 [23]. Until further high-quality evidence, including well-conducted randomized controlled trials, is available to demonstrate the definite role of AGMPs in spreading nosocomial infection, it is best to use the data available from past outbreaks to implement additional safeguards.

In this review, we performed a comprehensive literature search to present practical strategies (Table 2) to reduce the risk of nosocomial transmission when delivering AGMPs to patients with COVID-19. These suggestions are to be utilized in addition to the CDC recommendations available for proper personal protective equipment (PPE) for HCWs.

**Table 2:** Recommendations for providing respiratory care to COVID-19 patients

	Respiratory intervention	Evidence resource	Recommendation
1	Oxygen therapy	5 in vitro [24–28] 3 in vivo [29–31]	• Use nasal cannula and place a surgical/procedure mask on the patient's face • Avoid Venturi mask • Avoid nonrebreather mask unless it is filtered
2	High-flow nasal cannula	1 in vitro [32] 2 in vivo [15, 31]	• Proper nasal cannula fitting • Place a surgical/procedure mask over HFNC on the patient's face (Fig. 2)
3	Nebulization	2 in vitro [33, 34] 2 in vivo [22, 35]	• Use metered dosed inhaler with spacer when possible • Avoid using small volume nebulizer unless it is filtered (Fig. 3a, b) • Use nebulizer in line with HFNC or via ventilator
4	Lung expansion and airway clearance therapy*	3 in vivo [22, 35, 36]	• If using IPPB, place a filter between circuit and mask or mouthpiece, or on expiratory port • If possible, avoid cough inducing therapies such as intermittent percussive ventilation and cough assist • During high-frequency chest wall oscillation therapy, place a surgical/procedure mask on the patient's face
5	Non-invasive ventilation*	2 in vitro [37, 38] 2 in vivo [39, 40]	• Use tight fit oral mask without leaks, consider helmet or total face mask if available • Avoid using nasal mask • When using non-heated-wire single-limb circuit, place a filter between the non-vented mask and the expiratory port (Fig. 4a) • If humidification is required, heated wire single-limb circuit with filter placed at the expiratory port for non-invasive ventilator (Fig. 4b) or heated wire dual-limb circuits with critical care ventilator can be utilized
6	Intubation and Invasive ventilation*	1 in vitro [41] 4 in vivo [22, 39, 42, 43]	• During bag mask ventilation, place a filter between the mask and resuscitation bag (Fig. 5) • Most experienced provider performs intubation • Use video-laryngoscope • Rapid sequence intubation • Avoid breaking the ventilator circuit
7	Ventilator weaning		• Avoid cool aerosol for tracheostomy patient, instead use HME. If the patient needs frequent suctioning (more than once every hour), place an in-line suction catheter with T-piece connected to cool aerosol or heated humidification, the other end of T-piece connected to a filter (Fig. 6). Additionally, if the patient has cuffless tracheotomy, place a procedure mask on patient’s face • Avoid using T-piece trials. If needed, use the setup with a filter described above
8	Extubation*		• When removing the endotracheal tube, simultaneously turn off the ventilator • Avoid disconnecting ETT from the ventilator circuit before extubation to reduce spray of contaminated aerosols
9	Transport		• Place a filter between the artificial airway and the transport ventilator circuit • Use HME that has filter function (HME-F) • Consider clamping the ETT before disconnection from ventilator circuit
10	Bronchoscopy assist*	2 in vivo [44, 45]	• For spontaneously breathing patients, place a surgical mask on patient's face (Fig. 7a, b) • Use NIV mask with examination port for patients on NIV (Fig. 7d) • Use swivel adapter to insert bronchoscope for intubated patient (Fig. 7c)

*Abbreviations*: *HFNC* high-flow nasal cannula, *IPPB* intermittent positive pressure breathing, *HME* heat moisture exchanger, *ETT* endotracheal tube, *NIV* non-invasive ventilation

*Based on CDC guidelines, these procedures should ideally be performed in airborne infection isolation rooms

## Literature search strategy

**Fig. 1:**
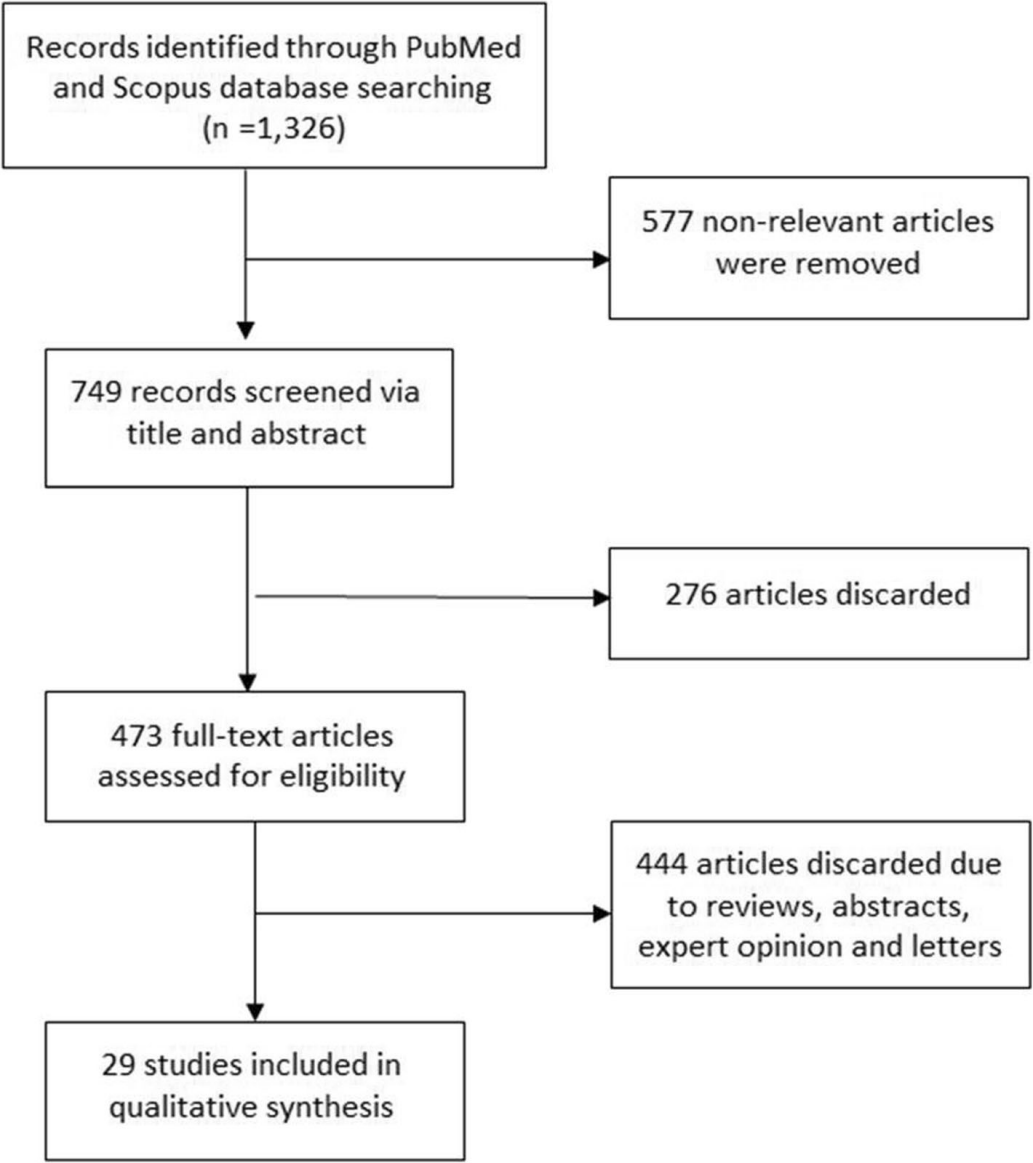
Flow diagram of the literature search

A literature search was performed via PubMed and Scopus databases using the following keywords: (“coronavirus” OR “COVID-19” OR “Severe Acute Respiratory Syndrome” OR “SARS” OR “Middle East Respiratory Syndrome” OR “MERS” OR “H1N1”) AND (“aerosol generating procedures” OR “nosocomial infection”). Publication types included systematic review, meta-analysis, randomized clinical trials, and observation studies. The study population involved HCWs providing respiratory care including AGMPs to patients infected with SARS, MERS, influenza A virus subtype H1N1 (H1N1), or COVID-19. In vitro studies investigating the role of exhaled air dispersion when providing AGMPs were also included. Published letters, book chapters, conference abstracts, and editorials were excluded. The literature search was limited to articles published until May 2020. The detailed selection process conducted is shown in Fig. 1.

## Literature findings and suggestions

A common clinical finding with COVID-19 is cough [7]. Coughing, speaking, laughing, and breathing have been associated with generation of bio-aerosols capable of carrying the virus [46, 47]. The bio-aerosols can range from 0.1 to 100 μm, and particles smaller than 1 μm have been reported to disperse to greater distances and remain airborne for several hours [5, 48, 49]. Large particles tend to settle directly on surfaces surrounding the patients, with reports of surface swabs testing positive across the patient’s room [5, 50]. A recent experimental study indicated that the COVID-19 virus can remain viable and infectious in aerosol for hours and on surface for days [51] and virus-laden aerosol deposition plays a role in surface contamination [4]. Some medical procedures that cause/irritate patients to cough or sneeze, such as bronchoscopy and nasal-pharyngeal suctioning, lead to the generation of bio-aerosols from patients. In contrast, other medical procedures do not “generate” bio-aerosols but increase the dispersion of bio-aerosols generated by infectious patients, such as NIV and high-flow nasal cannula (HFNC) oxygen therapy [5, 19]. Based on CDC guidelines, HCWs performing AGMPs should wear N95 or high-level respirators along with eye protection, gloves, and a gown. Furthermore, the number of personnel entering patient’s room during AGMPs should be limited and procedures should be ideally performed in an airborne infection isolation room [52].

### Oxygen therapy

Supplemental oxygen therapy is essential for patients with hypoxemic respiratory failure. While supplemental oxygen has not been shown to generate bio-aerosols, they may have a role in dispersing them. In an in vitro study using a human simulator with smoke (< 1 μm aerosol of solid particles) exhaled through airway, Hui et al. examined the exhaled air dispersion during oxygen delivery via nasal cannula. The results showed that exhaled air dispersion increased as oxygen flow was increased from 1 to 5 L/min and substantial exposure occurred within 1 m from the bed in a negative pressure ventilation room [24]. A substantial increase in lateral exhaled air dispersion is reported as the oxygen flows increased [25–27]. The same group of researchers using a similar model reported that both nonrebreather and air-entrainment masks increased exhaled air dispersion [25–27]. Exhaled air dispersion distance was further with the air-entrainment mask than simple and nonrebreather masks [53].

Placing a simple surgical mask on patient’s face has been reported to reduce the exhaled dispersion distance [28, 29] and the influenza A virus load [30] during a cough. Surgical masks and N95 masks are similarly effective at preventing influence virus exposure [30]. Placing either mask on a patient with confirmed COVID-19 can help reduce the dispersion of bio-aerosols [15]. Based on these findings, when a standard nasal cannula is used to deliver low-flow oxygen therapy, a surgical mask should be placed over the patient’s face. The air-entrainment mask should be avoided for patients with COVID-19, if possible. If higher delivered F_I_O_2_ is needed, a closed non-breather mask with a filter could be considered [54].

**Fig. 2:**
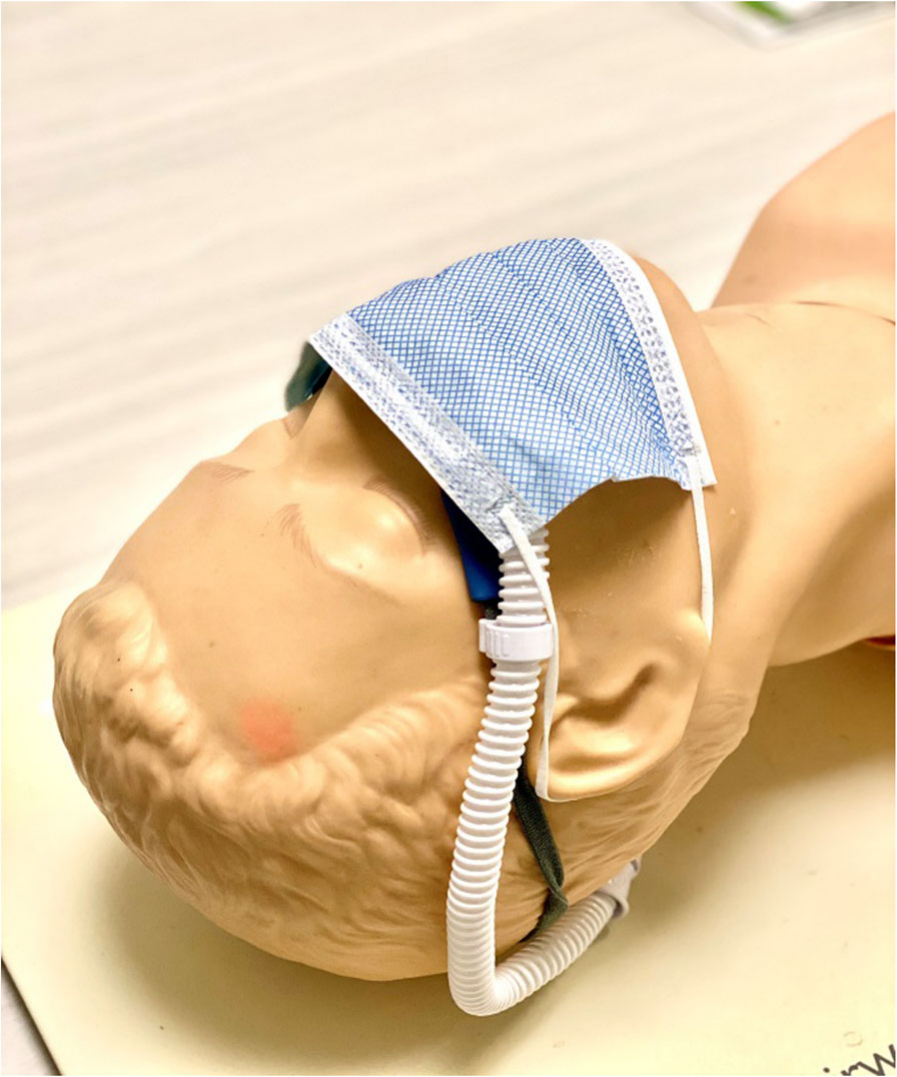
Wearing a surgical mask over high-flow high humidity nasal cannula

Oxygen delivery via HFNC has become widely used in patients with acute hypoxemic respiratory failure due to its benefits of meeting or exceeding patient inspiratory flow demand, reducing oxygen dilution, and washing out pharyngeal dead space [55]. HFNC has been shown to reduce the need for endotracheal intubation when compared to conventional oxygen delivery devices [56]. Two retrospective studies examining the effects of HFNC in patients with acute hypoxemic respiratory failure secondary to COVID-19 showed that HFNC was able to maintain adequate oxygenation and reduced the need for NIV and mechanical ventilation [32, 57]. Exhaled smoke dispersion, from a manikin during HFNC treatment, was shown to significantly increase with increased flow rate [58]. Interestingly, the dispersion distance from the HFNC at 60 L/min was shorter than an air-entrainment or nonrebreather mask [53]. A randomized controlled, crossover non-inferiority study trial reported no difference in gram-negative bacterial and total bacterial counts between HFNC at 60 L/min and simple oxygen mask at 8 L/min when air sample collection plates were placed at 0.4 or 1.5 m away from the patient [31]. It is important to note that a substantial increase of exhaled smoke dispersion was reported when the nasal cannula connection with patient nares was loose [58, 59]. Because of these findings, it is suggested that a surgical or procedure mask be worn by patients receiving HFNC (Fig. 2). Regular checks on the proper position and connection of the nasal cannula interface under the mask are also necessary.

### Nebulization

Aerosol therapy has been identified as a high-risk procedure for nosocomial transmission, due to its active generation of aerosol, which may carry viruses into the environment [19, 60]. Hui and colleagues found the maximum exhaled air dispersion distance was ≥ 0.45 m when a small volume jet nebulizer (SVN) was connected to a mask at a gas flow of 6 L/min [33]. This distance was even further than NIV at maximum settings (IPAP 18 cmH_2_O, EPAP 4 cmH_2_O), using the same study method [33]. Two clinical observational studies also found droplet counts significantly increased immediately after SVN started to generate aerosol, particularly, the aerosol/droplet count within small and medium size range 1–5 μm, when compared to the baseline level or other procedures including oxygen therapy and NIV [35] or bronchoscopy examination [44]. Nevertheless, the aerosol/droplets generated by a nebulizer may not contain a virus; however, if the nebulizer is contaminated, the aerosol can carry viruses to the surrounding environment. McGrath and colleagues [34] found that mass concentrations of aerosols/droplets were significantly reduced after placing a filter at the end of the mouthpiece for nebulizers. Therefore, if aerosol therapy is indicated for COVID-19 patients, SVN should be avoided unless filtered, and inhalers including metered dose inhaler (MDI) and dry power inhalers (DPIs) are preferred for spontaneous breathing patients who can tolerate their use without generating additional cough [61].

**Fig. 3:**
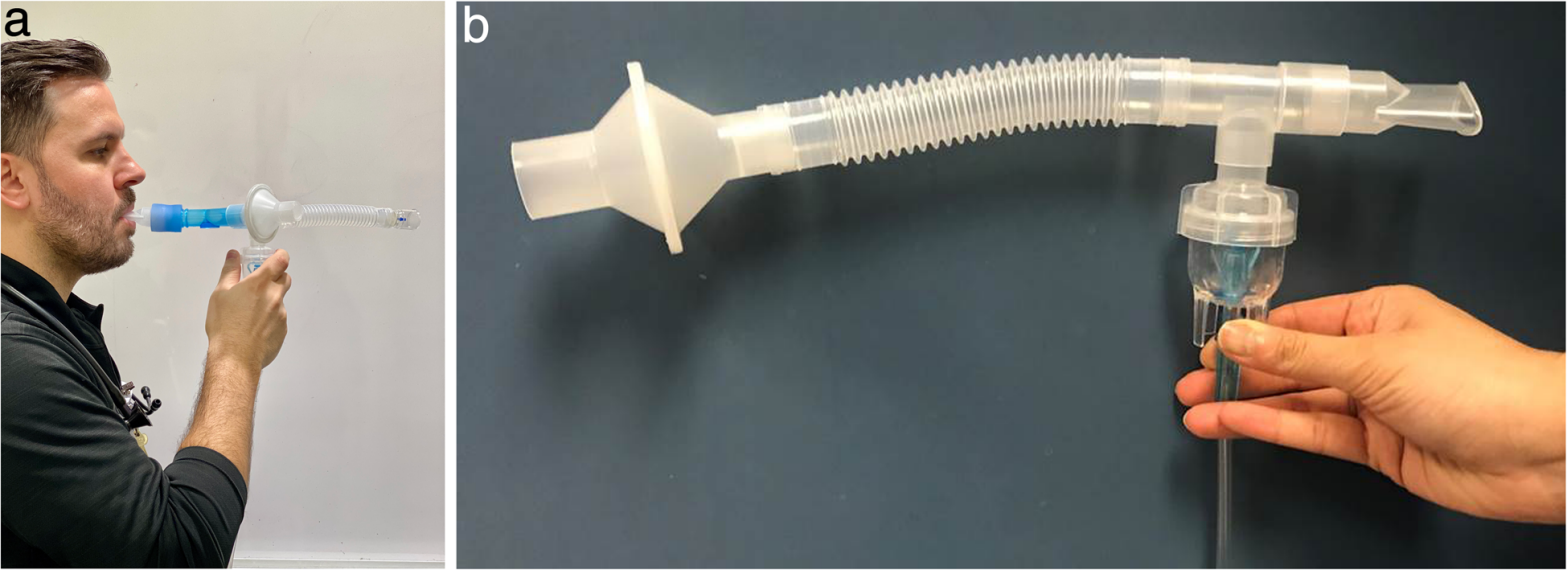
**a** SVN setup with filter and one-way valve. **b** SVN setup with a filter

With MDI, a spacer with one-way valve is suggested to reduce the need for coordination and to increase lung deposition [62]. If patients are unable to use MDIs or DPIs, or the required medication is only available in the form of a solution, such as antibiotics, antivirals, mucokinetics, or prostanoids, nebulizers via mouthpiece with a filter placed distal to the reservoir tubing (Fig. 3a and b) should be utilized. For patients who cannot tolerate a mouthpiece or require medication administered over a prolonged period of time, such as continuous bronchodilator for asthmatic patients [63] or inhaled epoprostenol for patients with pulmonary hypertension or hypoxemia [64, 65], in-line placement of a nebulizer with HFNC setup is recommended. This setup has two advantages: (1) more comfortable and better tolerated when compared to a mask or mouthpiece [63] and (2) a surgical mask to reduce the aerosol dispersion distance or aerosol mass concentration can be placed on the patient [53, 56]. When HFNC is utilized to deliver aerosol treatment, gas flow needs to be set relatively low if possible (10–20 L/min for adults and 0.25 L/kg/min for children), to improve the aerosol delivery efficiency [66, 67] and reduce the dispersion. Vibrating mesh nebulizers or valved T-pieces for jet SVNs can reduce the need to break the ventilator circuit when nebulization is provided during invasive ventilation.

### Lung expansion and airway clearance therapy

Little evidence is available regarding lung expansion therapy and nosocomial infection. Lung expansion therapy is designed to treat and prevent pulmonary atelectasis. Intermittent positive pressure breathing (IPPB) utilizes short-term positive pressure ventilation via mask or mouthpiece to promote lung expansion. Due to the risk of causing a cough response that might disperse bio-aerosols [21], IPPB should be used judiciously and with filters placed between the breathing circuit and the mask or mouthpiece.

The number of particles emitted by cough from an infected patient was greater than that from a recovered patient (*P* < 0.001) [36]. Bronchial hygiene therapies such as intermittent percussive ventilation and vibratory positive expiratory pressure irritate the airway causing the patient to cough forcefully, potentially emitting virus-laden aerosols. Placing a filter between these devices and patient’s mouth is suggested. When intermittent percussive ventilation is utilized, nebulization via its integrated nebulizer should be avoided as the filter placed between the device outlet and patient will capture aerosols. Additionally, high-frequency chest wall oscillation can be used for secretion clearance. In addition to HCWs wearing proper PPE, a surgical or procedure mask worn by patients receiving the therapy may be helpful. Overall, in patients with confirmed COVID-19, avoid the indiscriminate use of bronchial hygiene therapies that may not be clinically indicated [61].

### Non-invasive ventilation

NIV has been utilized in 10–50% of COVID-19 patients in published clinical reports [6–12]. Even though NIV delivered by helmet was effective in terms of reducing intubation rate and 90-day mortality rate among ARDS patients [68], its role in patients with severe ARDS remains controversial [69]. During the MERS outbreak, NIV was commonly used to treat acute hypoxic respiratory failure, but it had a high failure rate and was not associated with improved patient outcomes [70].

NIV produces a jet of exhaled gas through the exhalation port or leak from the connection of patient’s interface and ventilator, increasing dispersion distance of patient-generated bio-aerosol, and therefore  it is counted as an AGMP [20, 39]. Consequently, NIV should be used with caution for COVID-19 patients and additional modifications to minimize or reduce exhaled gas/aerosol dispersion are required.

In an experimental study, Hui et al. reported significant exhaled air dispersion within a 0.5-m radius of the human simulator receiving NIV and higher pressure settings increased the spread of exhaled air. However, the exhaled air dispersion is limited if the mask fit is appropriate [37]. When comparing helmet to total face mask, Hui et al. [38] in another study demonstrated that NIV application via a double-limb circuit ventilator with filters and a helmet with good seal was effective in reducing exhaled air dispersion. In contrast, NIV applied via a total mask through a single-limb circuit ventilator caused increased exhaled air dispersion [38]. Non-vented masks were shown to have less air dispersion as compared to vented mask [71].

**Fig. 4:**
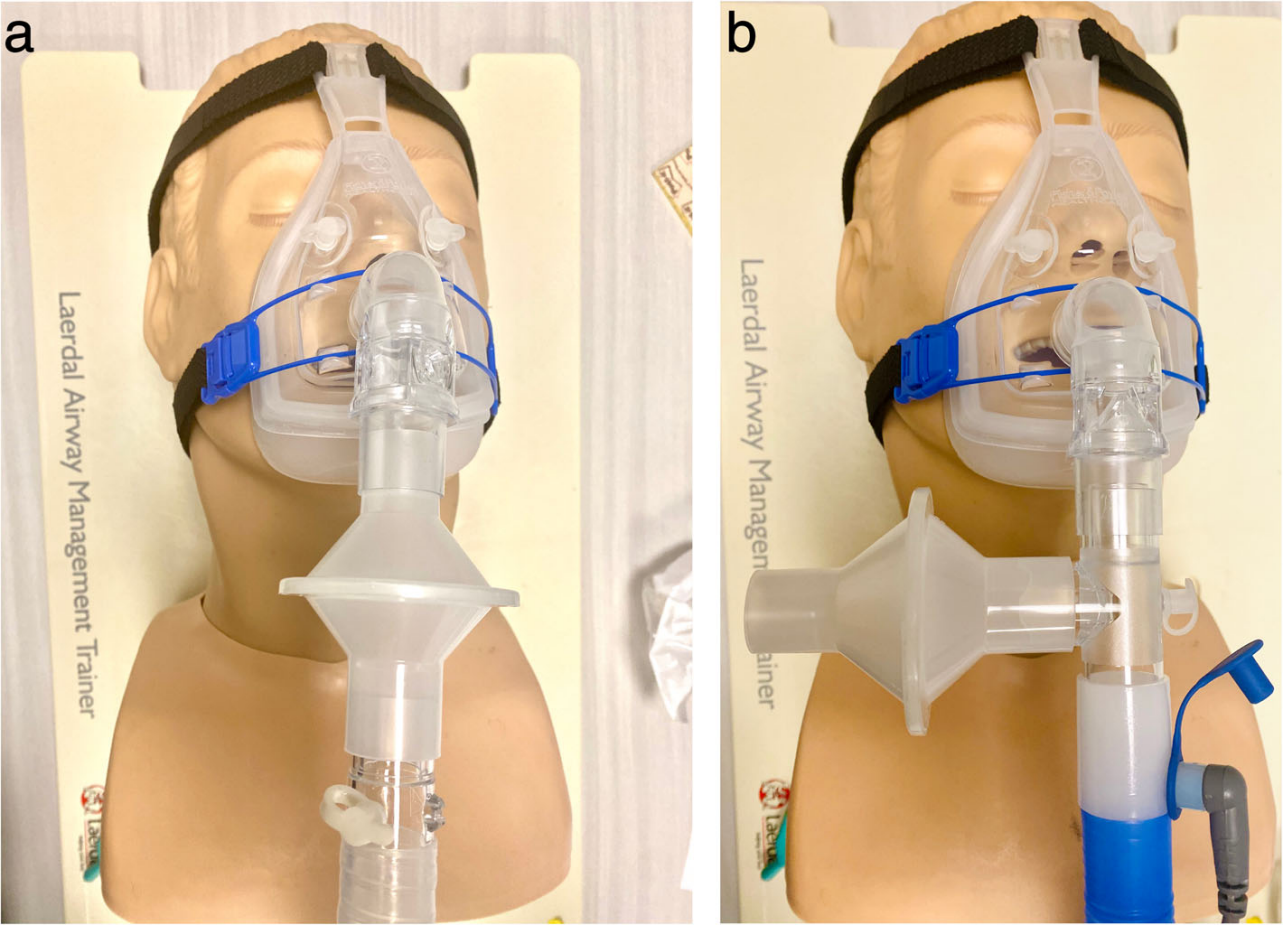
**a** Non-heated single limb ventilator circuit. **b** Heated single limb ventilator circuit

Thus, when vented masks are used, additional precautions for protection of the HCW may be appropriate. HCWs that are in close proximity to patients receiving NIV need to wear high respiratory personal protection including N95 or powered air-purifying respirator (PAPR). Secondly, the NIV circuits can be modified to place a filter. During the SARS outbreak, Cheung et al. demonstrated that a filter placed before the fixed exhalation port in the single-limb circuit was effective in reducing the incidence of nosocomial transmission among HCWs [40]. However, due to the lack of a control group, the results of this study should be interpreted cautiously. Additionally, Simonds et al. showed that modifying NIV circuit with a filter was effective in reducing the droplet counts [35]. Thus, a filter should be placed between the non-vented mask and the exhalation port to reduce environmental contamination of bio-aerosols (Fig. 4a). Notably, humidification should be avoided in this type of circuit as the viral  filter may capture water vapor in the circuit, resulting in occlusion for exhalation. If humidification is necessary, a modified exhalation port is needed to place a filter at the outlet (Fig. 4b). An alternative is using a dual-limb circuit ventilator with filters to deliver NIV. This would allow for both humidification and the reduction in exhaled gas/aerosol dispersion.

Regardless of interface/ventilator, the risk of a leak between the patient and the mask interface cannot be overlooked. Choosing an appropriate interface size and type, along with the appropriate circuits and ventilators, is crucial. A good fitting oral mask is preferred and avoid using a nasal mask for patients with COVID-19. If unable to get a good seal with an oral mask, consider using a total face mask or a helmet, if available.

### Intubation and mechanical ventilation

Clinicians who perform or assist in endotracheal intubation are directly exposed to patient’s lower airway where high concentrations of virus is accumulated. Additionally, in patients with an intact cough or gag reflex, intubation may increase exhaled air dispersion [72]. Therefore, intubation is considered high risk [73]. The risk of being infected when performing or assisting intubation (RR, 13.29; 95% CI, 2.99–59.04; *p* = 0.003) was found in the outbreak of SARS in a Canadian ICU [39]. Since then, high levels of PPE and negative pressure environments have been recommended to protect clinicians during intubation [74]. To reduce the exposure time to the SARS-CoV-2, the most experienced provider should perform intubation to avoid multiple attempts. Video-laryngoscope has shown to be useful when intubating patients with COVID-19 to increase the distance between the provider and the patient airway [74]. For a difficult airway, bronchoscopy is preferred to assist intubation, if a skilled provider is present [75]. Rapid sequence intubation is also recommended, in order to minimize cough during the procedure [42, 76]. Aerosol boxes [77] as well as protective shields made of glass [78] have been described as practical barriers to limit exposure to patient’s exhaled droplets during intubation. While potentially useful, a documented reduction in disease transmission has not been reported and concerns regarding adequate airway view and appropriate ergonomics during intubation have been raised [79].

**Fig. 5:**
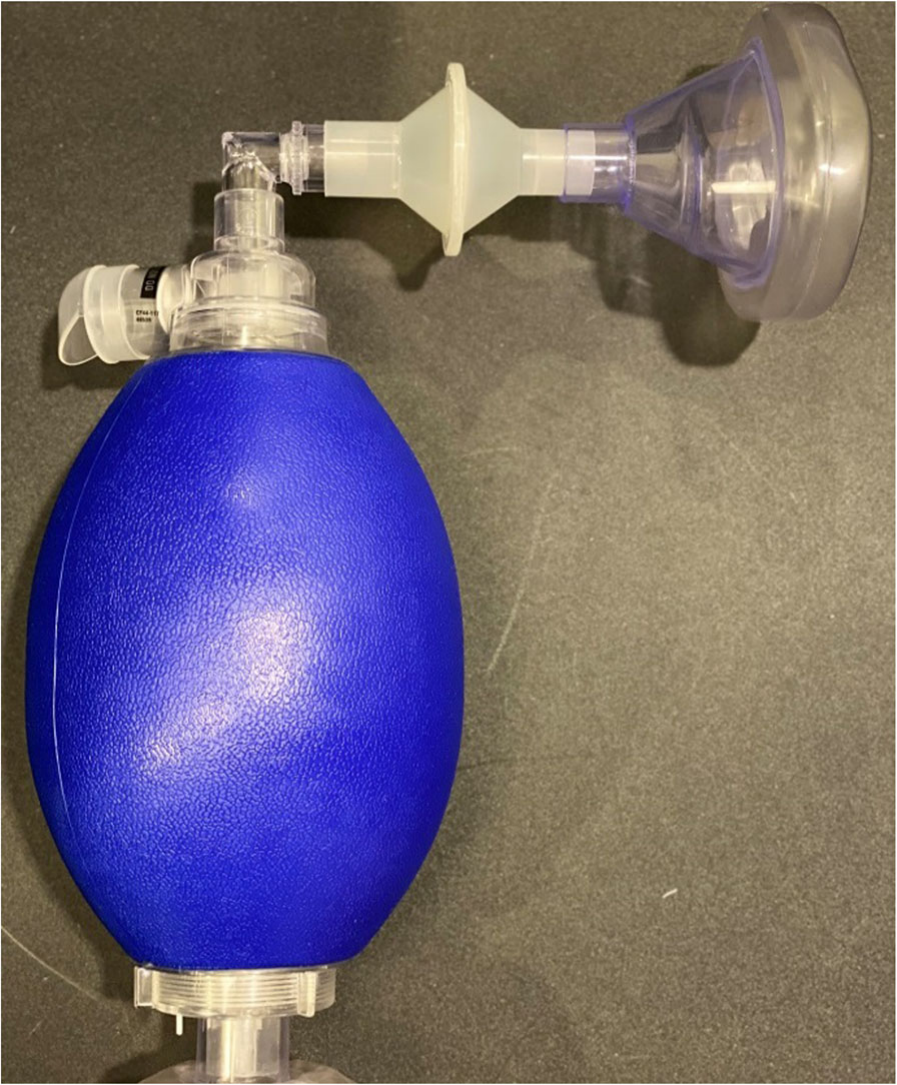
Resuscitation bag setup with a filter

Pre-oxygenation prior to intubation plays a crucial role in avoiding complications during intubation. Multiple randomized controlled trials have shown that the utilization of HFNC for pre-oxygenation can help reduce the incidence of hypoxemia during intubation [55, 80]. The cost-effectiveness and the high risk of transmission from high gas flows should be taken into consideration before using it for pre-oxygenation prior to intubation. The traditional method of using manual ventilation via resuscitator and mask for patients prior to intubation also has some risks. Exhaled gas dispersion distance has been shown to be 16–27 cm during manual ventilation, which is similar to the distance between clinicians and the patient’s airway [41]. Placing a filter between resuscitator and mask (Fig. 5) has been found to significantly reduce the exhaled gas dispersion distance [41, 43, 81].

For patients requiring mechanical ventilation via artificial airways, a ventilator with a dual-limb, heated wire circuit in conjunction with filters placed at the ventilator exhalation outlet is crucial [61, 73]. In addition, breaking the ventilator circuit connection should be limited and circuit changes should only be done when visibly soiled [82].

Approximately 8–13% mechanically ventilated patients receive tracheostomy to facilitate the long-term need for ventilatory support [83]. For patients with COVID-19, open tracheostomy is recommended over percutaneous dilational tracheostomy to reduce the risk of aerosol transmission [84]. Recently, Pichi et al. described standard steps to promote a safe and effective method when performing open tracheostomy in patients with COVID-19 [85]. Bertroche et al. [86] created a negative pressure cover to limit the exposure to the aerosols, but these methods need further investigation on the efficacy in reducing nosocomial infections.

When transporting a mechanically ventilated COVID-19 patient, it is suggested that a filter HME be placed between the artificial airway and the transport ventilator circuit [53]. Before pausing the ICU ventilator, consider clamping the endotracheal tube (ETT) to prevent derecruitment and minimize the spread of bio-aerosols when transitioning patients from the ICU ventilator to the transport ventilator [5, 87, 88]. When returning to the ICU, clamp the ETT and leave the filter connected to it to prevent accidental exposure. When ready for transition to the ICU ventilator, disconnect the bacteria filter and place the patient on the ventilator before unclamping ETT.

### Weaning and extubation

**Fig. 6:**
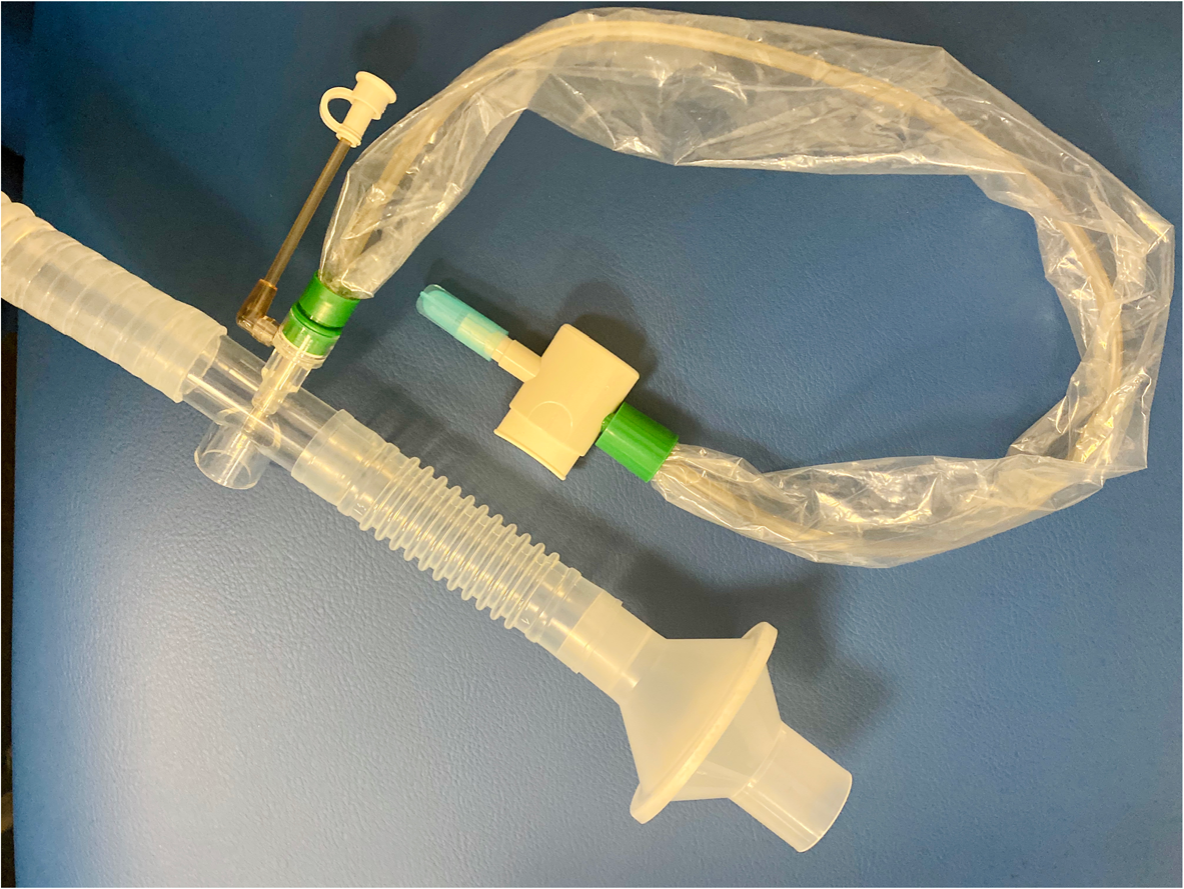
T-piece setup for tracheostomy patients

The most common methods to perform a spontaneous breathing trial are T-piece trial and pressure support ventilation (PSV). Subirà and colleagues [89] reported that successful extubation occurred in 82.3% of patients in the PSV group compared to 74.0% in the T-piece group (difference, 8.2%; 95% CI, 3.4–13.0%; *P* = 0.001). With these findings, in conjunction with the need to avoid opening patient’s airway to the environment, PSV is preferred for COVID-19 patients. When a T-piece is needed, HCWs should take safety precautions to minimize the exposure to a patient’s airway, such as using the in-line suction catheter’s T-piece with one end connecting humidified oxygen while the other end is connected to a filter (Fig. 6). This setup can also be applied for tracheostomy patients who are weaned from mechanical ventilation, particularly for patients who need frequent suctioning (more than once an hour), as this device keeps airway sealed and the filter protects HCWs during suctioning. However, the filter can be clogged as it captures water vapor; hence periodically checking and replacing the filter are necessary. A filter HME can also be used to provide passive humidity [90] while humidified oxygen via a tracheostomy mask should be avoided. Additionally, if the patient has a cuffless tracheostomy tube in place, a procedure mask on patient’s face may reduce bio-aerosol dispersion.

The process of extubation induces a cough reflex which may spread aerosols; therefore, it is imperative to use proper precautions when removing an ETT [91]. During extubation, it is important to maintain the connection of the ventilator circuit and suction catheter to the ETT, in order to avoid aerosol dispersion from the ventilator circuit. The extubation procedure should be performed by two HCWs. In an in vitro study, a clear plastic drape was shown to significantly reduce aerosol dispersion during the extubation process; however, the feasibility of this practice requires further investigation [92].

### Bronchoscopy assist

Bronchoscopy examination is considered an AGMP and may be related to an increased risk for transmission of infectious airborne particles [19]. Thompson et al. [45] found that bronchoscopy was associated with increased probability of aerosol generation and increased viral copies among different AGMPs for H1N1-positive patients. O’Neil et al [44] found an increase in particle concentration when a nebulized medication administration was performed before and after bronchoscopy, while bronchoscopy examination itself did not increase concentration compared to baseline.

According to the American Association for Bronchology and Interventional Pulmonology guidelines, bronchoscopy procedures are relatively contraindicated for patients with suspected or confirmed COVID-19 infections when less invasive diagnostic procedures are inconclusive [93]. Urgent bronchoscopy procedures should only be considered if intervention is deemed as lifesaving in patients with (1) massive hemoptysis, (2) benign or malignant severe airway obstruction, (3) suspicion of secondary infectious etiology, or (4) malignant condition that results in endobronchial obstruction. In the event a COVID-19 patient requires bronchoscopic intervention, it is recommended that the patient be placed in negative pressure isolation room and personnel should don appropriate droplet precaution PPE, including a powered air-purifying respirator or N95 mask [93].

**Fig. 7:**
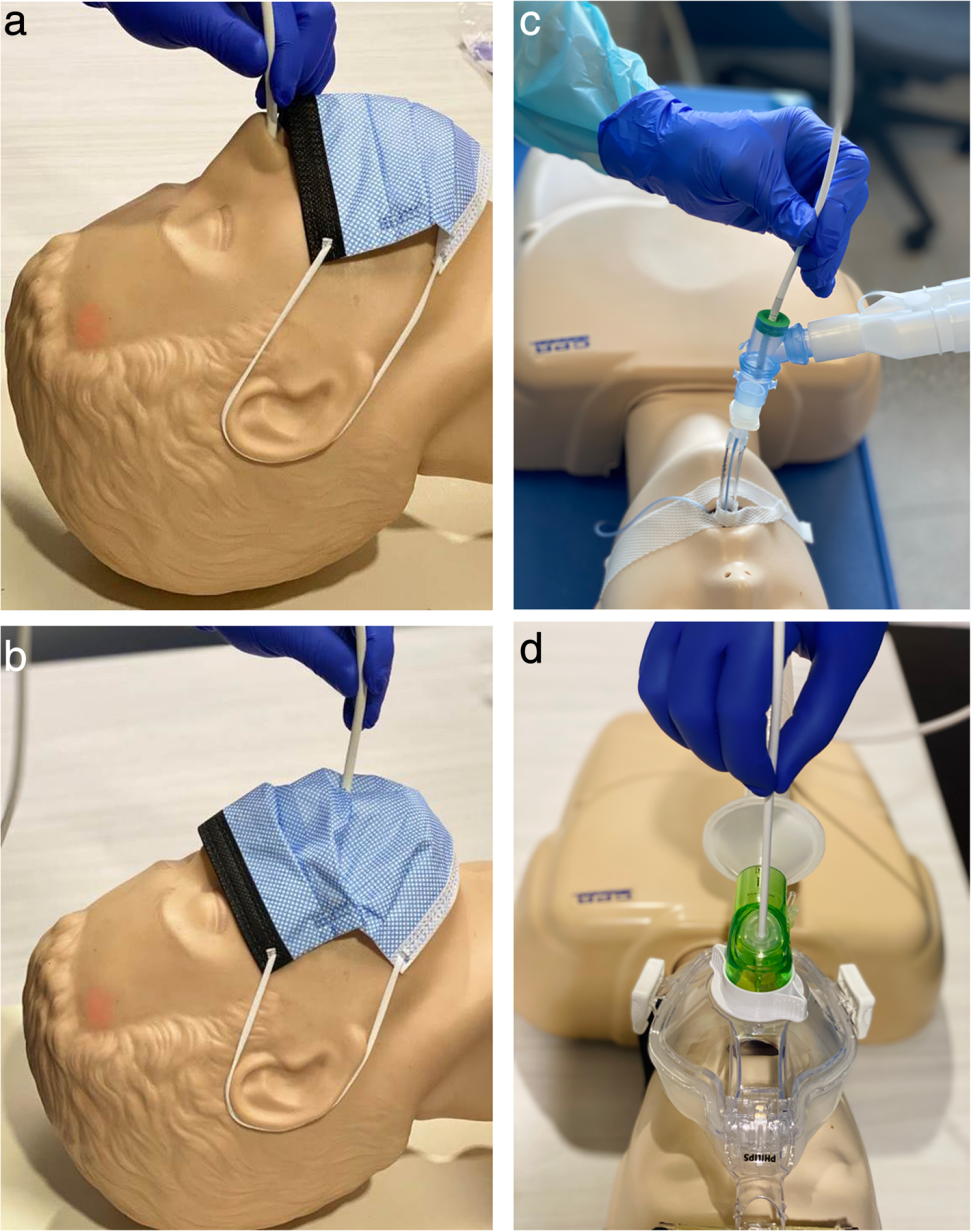
**a** Bronchoscope insertion via the nose. **b** Bronchoscope insertion via the mouth. **c** Bronchoscope insertion via the endotracheal tube. **d** Bronchoscope insertion via the NIV mask

Some additional precautions might also be considered to protect HCWs from exposure during bronchoscopy [94, 95]. (1) For spontaneously breathing patients, if the bronchoscope is inserted via the nares, a surgical or procedure mask should be placed to cover the face (Fig. 7a). If inserted via the mouth with a bite-block, a surgical or procedure mask with a small hole cut for bronchoscope insertion should be placed on the patient’s face (Fig. 7b). (2) For non-invasively ventilated patients, a special NIV mask with an examination port should be used (Fig. 7d). (3) For invasively ventilated patients, a swivel adapter should be used to facilitate the bronchoscope insertion and to maintain ventilation (Fig. 7c).

### Pulmonary function testing

Due to the risk of patient coughing and deep breathing during the procedure, pulmonary function testing is considered a platform for COVID-19 transmission. As a result, the American Thoracic Society recommends limiting testing to only those with immediate treatment needs [95] and, if possible, testing should only be performed upon symptom improvement and negative real-time polymerase chain reaction tests [96]. When performing pulmonary function tests, HCWs should adhere to strict infection control measures and use high specification disposable in-line viral/bacterial filters (minimum proven efficiency for high expiratory flow of 600 to 700 L/min) with the mouthpiece [97, 98]. When performing lung function testing in high-risk patients, the European Respiratory Society recommends that lung function testing should be limited to spirometry and diffusion capacity test. They also recommend the use of negative pressure rooms, when available [99]. Currently, there is limited data available if spirometry is an aerosol-producing procedure; therefore, HCWs should adhere to wearing full PPE [100].

## Conclusion

The frontline HCWs are at risk for contracting the COVID-19 disease when caring for patients and providing aerosol-generating procedures. Until further high-quality studies generate robust evidence, defining the precise nosocomial transmission risk associated with AGMPs, along with CDC’s recommended PPE guidelines, we propose additional respiratory protective measures that could reduce the nosocomial transmission of COVID-19 diseases to HCWs providing respiratory interventions.

## Data Availability

Not applicable

## Abbreviations

AGMP: Aerosol-generating medical procedure
ARDS: Acute respiratory distress syndrome
CDC: Centers for Disease Control and Prevention
CI: Confidence interval
COVID-19: Coronavirus disease
ETT: Endotracheal tube
H1N1: Influenza A virus subtype H1N1
HCW: Healthcare worker
HFNC: High-flow nasal cannula
HME: Heat moisture exchanger
HME-F: HME with HEPA filtering
ICU: Intensive care unit
IPPB: Intermittent positive pressure breathing
MERS: Middle East respiratory syndrome
NIV: Non-invasive ventilation
PPE: Personal protective equipment
PSV: Pressure support ventilation
RR: Relative risk
SARS: Severe acute respiratory syndrome
SVN: Small volume nebulizer
